# Relationship between health-related quality of life and environmental parenting among Korean parents of children and adolescents: the mediating effect of environmental health behavior in a cross-sectional study

**DOI:** 10.4069/whn.2026.03.04

**Published:** 2026-03-31

**Authors:** Hae Kyung Jo, Hyun Kyoung Kim

**Affiliations:** 1 Department of Nursing, Jeonju University, Jeonju, Korea; 2 Department of Nursing, Kongju National University, Gongju, Korea

**Keywords:** Environmental health, Health behavior, Parents, Quality of life

## Abstract

**Purpose:**

Environmental parenting refers to parental practices that aim to minimize children’s exposure to environmental hazards. This cross-sectional study examined the relationships among health-related quality of life (HRQoL), environmental parenting, and environmental health behavior among parents of children and adolescents.

**Methods:**

This correlational study was conducted between September and October 2024 and targeted parents with children aged 0 to 18 years. A total of 212 parents from three cities in Korea were recruited using convenience sampling and completed face-to-face questionnaires assessing HRQoL, environmental parenting, and environmental health behaviors. Pearson correlation coefficients were calculated, and mediation analysis was conducted using multiple regression with the PROCESS macro, applying 95% bias-corrected bootstrap confidence intervals based on 5,000 resamples.

**Results:**

The total effect of HRQoL on environmental parenting was significant (B=–1.92, *p*=.032). HRQoL was also significantly associated with environmental health behavior (B=–1.59, *p*=.028), and environmental health behavior was significantly associated with environmental parenting (B=0.91, *p*<.001). However, the direct effect of HRQoL on environmental parenting was not significant (B=–0.45, *p*=.446). Environmental health behavior fully mediated the relationship between HRQoL and environmental parenting (B=–1.46; 95% confidence interval, –2.79 to –0.14).

**Conclusion:**

These findings highlight a pathway linking HRQoL to environmental parenting through environmental health behavior among Korean parents. Accordingly, interventions that promote parental health behaviors may strengthen environmental parenting practices, particularly in contexts characterized by higher environmental health behavior and HRQoL.

## Introduction

Environmental parenting refers to parental practices that reduce children’s exposure to environmental hazards and promote environmentally responsible behaviors within the household. This concept has gained increasing attention as children and adolescents encounter environmental toxins through multiple exposure pathways, including dust, food, and other household or community sources [1]. Because children aged 0 to 18 years have higher respiratory rates, developing organ systems, and proportionally greater exposure per body weight than adults, global environmental changes pose heightened risks for this population [2]. These threats contribute to adverse outcomes, including endocrine-related disorders such as diabetes, obesity, thyroid dysfunction, asthma, reproductive abnormalities, and neurodevelopmental conditions [3]. Protecting children from these hazards depends largely on the everyday decisions made by parents. Environmental parenting therefore represents an important behavioral domain through which children’s exposure risks may be mitigated [4].

Parental interventions play a central role in shaping children’s cognitive, emotional, and social development, and early learning experiences within the family provide a foundation for long-term growth [5]. In addition to general parenting practices, parents also protect children from environmental risks by identifying hazardous exposures, evaluating toxin-related threats, and adopting preventive strategies [6,7]. Within this context, environmental parenting has emerged as an important construct in child health research.

Health-related quality of life (HRQoL) reflects individuals’ physical, psychological, and social well-being. Higher levels of parental HRQoL have been consistently associated with greater engagement in health-promoting behaviors [7]. Individuals with higher subjective well-being tend to adhere more closely to healthy practices, including maintaining a balanced diet, engaging in regular physical activity, avoiding alcohol, and not smoking [8]. Parents who actively engage in these behaviors also influence their children’s values, lifestyle habits, and worldview, thereby encouraging environmentally oriented parenting practices [9]. Environmental health behavior may therefore influence environmental parenting, as parents’ own behaviors often extend to child-focused environmental protection [10]. In addition, parents with more favorable socioeconomic circumstances may be able to provide greater access to environmentally friendly products, healthier foods, and enriched educational opportunities, thereby linking higher social well-being to stronger environmental parenting [11]. Furthermore, physically and mentally healthy parents may help children minimize environmental health risks by preventing or reducing exposure to potential hazards [12]. Collectively, these pathways suggest that HRQoL may influence environmental parenting both directly and indirectly through environmental health behaviors.

**Figure 1.**
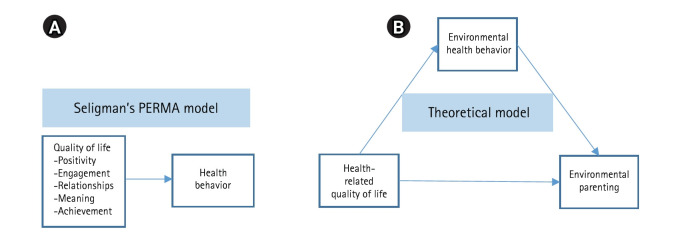
Conceptual framework according to the PERMA (Positive Emotion, Engagement, Relationships, Meaning, and Accomplishment) model. (A) PERMA Model [13] (B) Theoretical model of this study.

Grounded in Seligman’s PERMA (Positive Emotion, Engagement, Relationships, Meaning, and Accomplishment) model, which conceptualizes well-being as comprising emotional, cognitive, and relational resources [13], it is theoretically plausible that higher HRQoL enhances parents’ motivation and self-regulatory capacity to engage in consistent environmental health behaviors. These behaviors, in turn, may support environmentally responsible parenting. However, the sequential relationships among HRQoL, environmental health behavior, and environmental parenting have been more frequently proposed theoretically than empirically examined. Accordingly, examining whether environmental health behavior mediates the association between HRQoL and environmental parenting represents an important objective of the present study and addresses a meaningful gap in the literature (Figure 1).

This study aimed to examine the mediating effect of environmental health behavior on the relationship between HRQoL and environmental parenting among parents raising children aged 0 to 18 years. Specifically, the objectives were to (1) describe participants’ general characteristics, HRQoL, environmental health behavior, and environmental parenting; (2) examine the correlations among HRQoL, environmental health behavior, and environmental parenting; and (3) determine whether environmental health behavior mediates the relationship between HRQoL and environmental parenting. The hypotheses were as follows: (1) HRQoL will be positively associated with environmental health behavior; (2) environmental health behavior will be positively associated with environmental parenting; (3) HRQoL will be positively associated with environmental parenting; and (4) environmental health behavior will mediate the relationship between HRQoL and environmental parenting.

## Methods

**Ethics statement:** This study was approved by the Institutional Review Board of Jeonju University (No. jjIRB-231214-HR-2024-0905). Informed consent was obtained from all participants. All personal information collected through the survey was fully anonymized to ensure privacy and confidentiality.

### Study design

This descriptive correlational study employed a cross-sectional survey to examine the mediating role of environmental health behavior in the association between HRQoL and environmental parenting (Figure 1). The study was reported in accordance with the Strengthening the Reporting of Observational Studies in Epidemiology (STROBE) guidelines [14].

### Participants

The participants in this study were parents of children and adolescents aged 0 to 18 years residing in Korea. Participants were recruited through convenience sampling from public health centers, churches, community centers, schools, and educational facilities located in the cities of Milyang, Gongju, and Cheonan. Gongju is classified as a small-to-medium-sized city, whereas Cheonan is considered a medium-to-large city in Korea. Eligible participants were required to be at least 20 years of age, actively raising children younger than 18 years, and proficient in Korean. Individuals were excluded if they were currently hospitalized for health conditions or had difficulty understanding the purpose or content of the study. The required sample size for the regression analysis was estimated using G*Power ver. 3.1.9.7 (Heinrich Heine University Düsseldorf). Based on the guidelines proposed by Fritz and MacKinnon [15], an effect size of 0.08 was selected by averaging the small effect size of 0.14 reported by Fritz and MacKinnon [15] with the effect size of 0.03 reported in previous studies examining quality of life and health behavior [8]. Assuming an alpha level of 0.05 and statistical power of 0.95, the minimum required sample size was calculated to be 197 participants. To account for a potential nonresponse or exclusion rate of approximately 10%, 217 responses were initially collected. After excluding five incomplete questionnaires, data from 212 participants were included in the final analysis, corresponding to a response rate of 97.6%.

### Measurements

#### Environmental parenting

Environmental parenting was measured using the environmental parenting scale (EPS) developed by Jo and Kim [16]. The EPS consists of 21 items organized into four subdomains: hygiene management (seven items), natural product use (seven items), prevention of toxin exposure (four items), and protection from radiation (three items). Responses are recorded on a 5-point Likert scale ranging from 1 (not at all) to 5 (strongly agree). Higher total scores (possible range, 21–105) indicate greater engagement in environmental parenting practices. In the original study, the Cronbach’s α for the EPS was .93 [16]. In the present study, Cronbach’s α was .92.

#### Health-related quality of life

HRQoL was measured using the EuroQol 5-Dimension 3-Level (EQ-5D-3L), a standardized health status instrument developed by the EuroQol Group [17] and adapted for the Korean population by the Korea Disease Control and Prevention Agency [18]. The instrument assesses five dimensions—mobility, self-care, usual activities, pain/discomfort, and anxiety/depression—each measured with a single item using a three-level response system (1=no problems, 2=some problems, 3=extreme problems). Although the original instrument is typically used to calculate a preference-weighted utility index, this study instead summed responses across the five dimensions to generate a composite score representing overall health burden (possible range, 5–15), with higher scores indicating poorer HRQoL. This summed-score approach was used to examine relative differences in perceived health status within a community-based sample rather than preference-based health valuation [19]. Test-retest reliability reported in previous studies ranged from .86 to .90 [17], whereas the internal consistency reliability in the present study was .75.

#### Environmental health behavior

Environmental health behavior was measured using the Personal Environmental Health Behavior (PEHB) scale [20]. The PEHB is a 17-item instrument developed in Korean that evaluates personal environmental health practices across four subdomains: lifestyle (seven items), personal goods (four items), food (three items), and dust management (three items). Responses are rated on a 5-point Likert scale ranging from 1 (not at all) to 5 (strongly agree). Higher scores (possible range, 17–85) indicate greater engagement in environmental health behaviors. In the original study, the PEHB demonstrated a Cronbach’s α of .90 [20]. In the present study, Cronbach’s α was .92.

#### General characteristics

The following general characteristics were collected: age (year), gender (man/woman), marital status (married/unmarried), and number of children.

### Data collection

Data collection was conducted between September and October 2024. Researchers visited institutions that had agreed to participate after receiving study information distributed during recruitment. The researchers met with the heads of these institutions to explain the study objectives, data collection schedule, and research procedures, and obtained permission to proceed. Participants completed the questionnaires in person, and the self-administered surveys were collected on site. Completion of the questionnaire required approximately 10 to 15 minutes. Participants received a small gift valued at 6,000 Korean won (approximately 5 US dollars) as compensation for participation.

### Data analysis

Data were analyzed using IBM SPSS ver. 25.0 (IBM Corp., Armonk, NY, USA). Descriptive statistics were calculated to summarize participant characteristics and study variables. Pearson correlation coefficients were used to examine bivariate associations among environmental parenting (EPS), HRQoL (EQ-5D-3L), and environmental health behavior (PEHB). Mediation analysis was conducted to estimate the total, direct, and indirect effects of environmental health behavior on the relationship between parental HRQoL (EQ-5D-3L) and environmental parenting (EPS) using the PROCESS macro (ver. 3.5.3, Model 4). Bootstrapping with 5,000 resamples was used to estimate the indirect effect and to generate 95% bias-corrected confidence intervals [21].

## Results

### General characteristics

The mean age of participants was 45.42 years (SD=5.76 years), with ages ranging from 23 to 63 years. Among the participants, 136 (64.1%) were women and 76 (35.9%) were men. The mean number of children was 2.11 (SD=0.65). The mean EPS score was 79.23 (SD=13.25), indicating a moderate level of environmental parenting. The mean EQ-5D-3L score was 5.70 (SD=1.00), indicating relatively favorable HRQoL. The mean PEHB score was 62.25 (SD=10.80), reflecting a moderate level of environmental health behavior (Table 1).

### Correlations between environmental parenting, health-related quality of life, and environmental health behavior

A weak but statistically significant negative correlation was observed between EPS and EQ-5D-3L (r=–.15, *p*=.030). A similarly weak negative correlation was found between EQ-5D-3L and PEHB (r=–.15, *p*=.034). In contrast, EPS was strongly and positively correlated with PEHB (r=.75, *p*<.001) (Table 2).

### The mediating effect of environmental health behavior in the relationship between health-related quality of life and environmental parenting

Before testing the mediating effect, the assumptions for regression analysis were examined. The Durbin-Watson statistic was 1.69, indicating no serious autocorrelation. Cook’s distance values ranged from .00 to .13, and none exceeded the threshold of 1.00, suggesting the absence of influential outliers. Multicollinearity diagnostics also supported model adequacy: variance inflation factors ranged from 1.03 to 1.11, all well below the threshold of 10.00, and tolerance values exceeded 0.10. Collectively, these findings indicate that the data satisfied the assumptions required for regression analysis.

**Figure 2.**
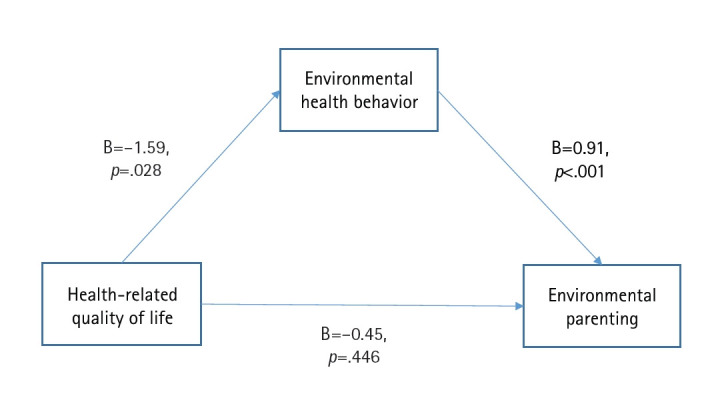
Mediating effect of environmental health behavior on the relationship between health-related quality of life and environmental parenting.

The total effect of EQ-5D-3L on EPS was significant (B=–1.92, *p*=.032). The effect of EQ-5D-3L on PEHB was also significant (B=–1.59, *p*=.028). In addition, PEHB was significantly associated with EPS (B=0.91, *p*<.001). However, the direct effect of EQ-5D-3L on EPS was not significant (B=–0.45, *p*=.446). A significant indirect effect of EQ-5D-3L on EPS through PEHB was observed (B=–1.46; 95% confidence interval, –2.79 to –0.14) (Table 3, Figure 2).

## Discussion

**Figure 3.**
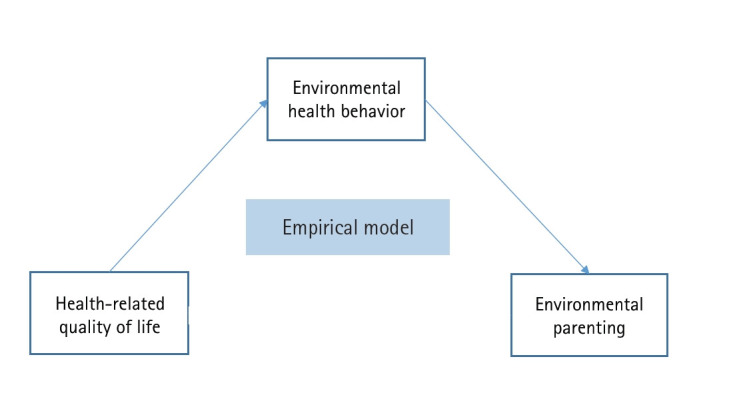
Empirical model for the study.

The findings of this study demonstrate relationships among environmental parenting, HRQoL, and environmental health behavior among Korean parents. These results support the view that individuals with better HRQoL may be more likely to adopt environmentally oriented behaviors as part of managing daily life and maintaining a sense of control [22]. In addition, the findings provide empirical support for Seligman’s PERMA model [13], according to which greater well-being enhances self-regulation, resilience, and motivation for adaptive behaviors. Consistent with this framework, the present study indicates that parents with better HRQoL were more likely to engage in environmental health behaviors (Figure 3). The observed association between HRQoL and environmental health behavior suggests that individuals reporting better quality of life also tend to report greater engagement in environmental behaviors [23], potentially reflecting shared psychosocial resources. This finding may have implications for intervention development by suggesting that efforts to strengthen HRQoL could be accompanied by support for environmental health behavior. Better quality of life may also be associated with improved emotional regulation, which could enable individuals to engage more consistently in purposeful health-related activities.

Active and problem-focused coping with stress has been associated with better well-being. Previous research suggests that optimistic views of the future may motivate young people to align their personal interests with broader social goals, thereby strengthening their sense of meaning in life [24]. In this context, individuals who perceive greater control over their environment may be more likely to report positive behaviors, including proactive environmental actions. These patterns may help explain why individuals with higher HRQoL also report more environmentally responsible behaviors. Overall, these associations suggest that greater well-being tends to co-occur with stronger engagement in sustainable and health-promoting environmental practices [25].

Furthermore, the strong positive association between environmental health behavior and environmental parenting indicates that environmentally oriented personal behaviors are closely related to parenting practices. This finding suggests that individuals who report environmentally friendly behaviors also tend to incorporate those behaviors into their parenting. It also underscores the importance of promoting environmental health behavior at the individual level, as such behaviors may extend to parenting practices and may help foster a culture of environmental responsibility within families. Individuals who engage in positive environmental health behaviors may therefore be more likely to practice active environmental parenting [26].

The strength and consistency of this association further suggest that environmentally conscious behavior co-occurs with environmentally responsible parenting practices, possibly through the modeling and prioritization of ecological values within family life. Collaborative initiatives involving educators and parents have been reported to effectively promote sustainable behaviors among children by creating shared learning experiences that influence both children and their parents [27]. By actively participating in activities that foster connectedness to nature, parents may not only model sustainable practices but also strengthen their relationships with their children, which may in turn encourage the adoption of environmentally friendly behaviors at home [28].

However, the direct relationship between HRQoL and environmental parenting was not statistically significant. Instead, the indirect effect through environmental health behavior appears to play a more important role. This finding suggests that improving HRQoL alone may not necessarily lead to stronger environmental parenting practices unless it is accompanied by support for specific environmental health behaviors. Therefore, initiatives designed to promote environmental parenting may benefit from prioritizing environmental health behavior, particularly among individuals with differing levels of HRQoL. Parents play an important role in shaping and promoting healthy behaviors in their children [29]. Consistent with this interpretation, a longitudinal cohort study found that higher subjective well-being predicted healthier behaviors, including reduced smoking and alcohol consumption, a healthier diet, and greater physical activity over a 9-year follow-up period [8]. Taken together, these findings suggest that HRQoL may be linked to environmental parenting primarily through environmental health behavior rather than through a direct pathway. Public health programs may therefore consider tailoring strategies according to individuals’ HRQoL levels. Although individuals with higher HRQoL may be more likely to engage in environmental behaviors, those with lower HRQoL may face greater barriers. Providing supportive opportunities for both groups may encourage environmental action while also enhancing well-being.

This study has several limitations. First, the use of convenience sampling may limit the generalizability of the findings to broader populations. Second, the wide age range of the children (0–18 years) may have introduced heterogeneity in parenting practices. Third, the substantial correlation between environmental health behavior and environmental parenting suggests that the two scales may reflect partially overlapping behavioral domains. Fourth, the cross-sectional design precludes any inference about the direction of associations or causality among the variables. Although significant associations were identified, it is not possible to determine whether one variable influenced another or whether unmeasured factors affected the observed relationships. Fifth, because the PEHB scale was originally developed to measure women’s personal environmental health behaviors, its application to a mixed-gender parent sample may reduce measurement validity for fathers, who comprised 35.9% of the sample. Finally, cultural and environmental differences may influence the relationships among HRQoL, environmental health behavior, and environmental parenting; future studies should therefore include more diverse populations to improve generalizability.

In conclusion, environmental health behavior fully mediated the relationship between HRQoL and environmental parenting, suggesting that environmental behaviors may represent an important pathway linking quality of life to parenting practices. These findings underscore the importance of interventions that not only support HRQoL but also actively encourage environmental health behaviors in order to strengthen environmental parenting. Promoting proactive environmental health behaviors may help foster more environmentally conscious parenting practices, with potential benefits for both family well-being and broader environmental outcomes. The findings of this study may inform the development of public health interventions and family-centered environmental education programs. Overall, promoting environmental health behaviors appears to be a promising strategy for supporting environmental parenting.

## Figures and Tables

**Table 1.** Descriptive statistics of participants (N=212)

Variables	Categories	Value	Possible range
Age (year)		45.42±5.76 (23–63)	
Gender	Woman	136 (64.1)	
	Man	76 (35.9)	
Marital status	Married	199 (93.8)	
	Unmarried	13 (6.2)	
No. of children		2.11±0.65 (1–4)	
Environmental parenting		79.23±13.25 (23–105)	21–105
Health-related quality of life		5.70±1.00 (5–10)	5–15
Environmental health behavior		62.25±10.80 (34–85)	17–85

Values are presented as mean±SD (range) or n (%).

**Table 2.** Correlations among environmental parenting, health-related quality of life, and environmental health behavior (N=212)

Variables	r (*p*)	
	Environmental parenting	Health-related quality of life
Environmental parenting	1	
Health-related quality of life	–.15 (.030)	1
Environmental health behavior	.75 (<.001)	–.15 (.034)

**Table 3.** Mediating effect of EHB between HRQoL and EP (N=212)

Variables	Effect	Label	B	SE	95% CI	Z	*p*
HRQoL-EP	Direct	c	–0.45	0.60	–1.64 to 0.72	–0.76	.446
HRQoL-EHB	Direct	a	–1.59	0.72	–3.02 to –0.17	–2.19	.028
EHB-EP	Direct	b	0.91	0.05	0.80 to 1.02	14.45	<.001
HRQoL-EHB-EP	Indirect	a×b	–1.46	0.67	–2.79 to –0.14	–2.17	.030
Total		c+a×b	–1.92	0.89	–3.68 to –0.17	–2.15	.032

CI: Confidence interval; EHB: environmental health behavior; EP: environmental parenting; HRQoL: health-related quality of life. Label: a, HRQoL→EHB; b, EHB→EP; c, HRQoL→EP.
